# Supplementary material for: SIRT3 Acts as a Positive Autophagy Regulator to Promote Lipid Mobilization in Adipocytes via Activating AMPK
Source: Int J Mol Sci. 2020 Jan 7;21(2):372. doi: 10.3390/ijms21020372 (PMC7013837; doi:10.3390/ijms21020372)
Supplement: Supplementary file 1 [file ijms-21-00372-s001.pdf]

Supplementary Information

SIRT3 acts as a positive autophagy regulator to promote lipid mobilization in adipocytes via activating AMPK

Tian Zhang[1], Jingxin Liu[1], Qiang Tong[2], Ligen Lin[1,*]

1 State Key Laboratory of Quality Research in Chinese Medicine, Institute of Chinese Medical Sciences, University of Macau, Macau, China; yb67520@um.edu.mo (T.Z.); jingxinliu@vip.sina.com (J.L.)

2 Children's Nutrition Research Center, Baylor College of Medicine, Houston, Texas, United States of America; qtong@bcm.edu

* Correspondence: ligenl@um.edu.mo; Address: 6044 Research Building N22, University of Macau, Avenida da Universidade, Taipa, Macau 999078, China; Tel.: +853-88228041 (L.L.)

Supplementary Table S1 siRNA sequence used in the current study.

siRNA target	Sense	Anti-sense	Catalog No.
Atg5	GCUACCCAGAUAACUUUCUTT	AGAAAGUUAUCUGGGUAGCTT	#1080
LAMP-2A	GCCGUUCAGUCCAAUGCAUTT	AUGCAUUGGACUGAACGGCTT	#215
AMPKα1	GCCGACCCAAUGAUAUCAUTT	AUGAUAUCAUUGGGUCGGCTT	#1250
AMPKα2	GCAGUGGCUUAUCAUCUUATT	UAAGAUGAUAAGCCACUGCTT	#1059
AMPKβ1	GCCAGCUUGGCACAGUUAATT	UUAACUGUGCCAAGCUGGCTT	#610
AMPKβ2	CCAGUCAGCUUGGAACAAUTT	AUUGUUCCAAGCUGACUGGTT	#608

Supplementary Table S2 Antibodies used for Western blots.

Antibody	Source	Vendor	Catalog No.
acetylated- lysine	Mouse	Protein Technology	#9441
AMPKα	Rabbit	Cell Signaling Technology	#2532
AMPKα1	Rabbit	Cell Signaling Technology	#5832
AMPKα2	Rabbit	Cell Signaling Technology	#2757
AMPKβ1	Rabbit	Cell Signaling Technology	#12036
AMPKβ2	Rabbit	Cell Signaling Technology	#4148
p-AMPKα (Thr172)	Rabbit	Cell Signaling Technology	#50081
Atg5	Rabbit	Cell Signaling Technology	#12994
Beclin1	Rabbit	Cell Signaling Technology	#3495
HSC70	Mouse	Cell Signaling Technology	10654-1-AP
LAMP-2A	Rabbit	Cell Signaling Technology	10397-1-AP
LC3	Rabbit	Cell Signaling Technology	#12741
PLIN1	Mouse	Cell Signaling Technology	#9349
p62	Rabbit	Sigma-Aldrich	P0067
SIRT3	Rabbit	Protein Technology	10099-1-AP
ULK1	Rabbit	Cell Signaling Technology	#8054
p-ULK1 (Ser555)	Rabbit	Cell Signaling Technology	#5869
GAPDH	Rabbit	Santa Cruz Biotechnology	sc-25778

Supplemental Fig. S1

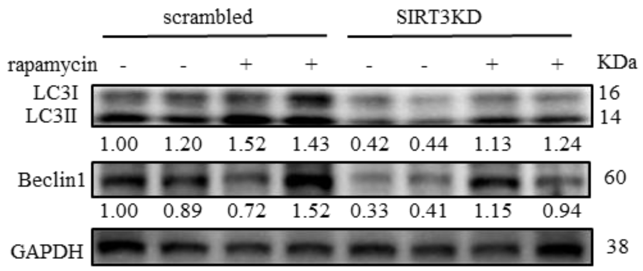

Supplemental Fig. S1. SIRT3KD and scrambled 3T3-L1 cells were treated with or without rapamycin for 16 h. Expression of autophagy related proteins was analyzed by Western blots. GAPDH was used as a loading control.

Supplemental Fig. S2

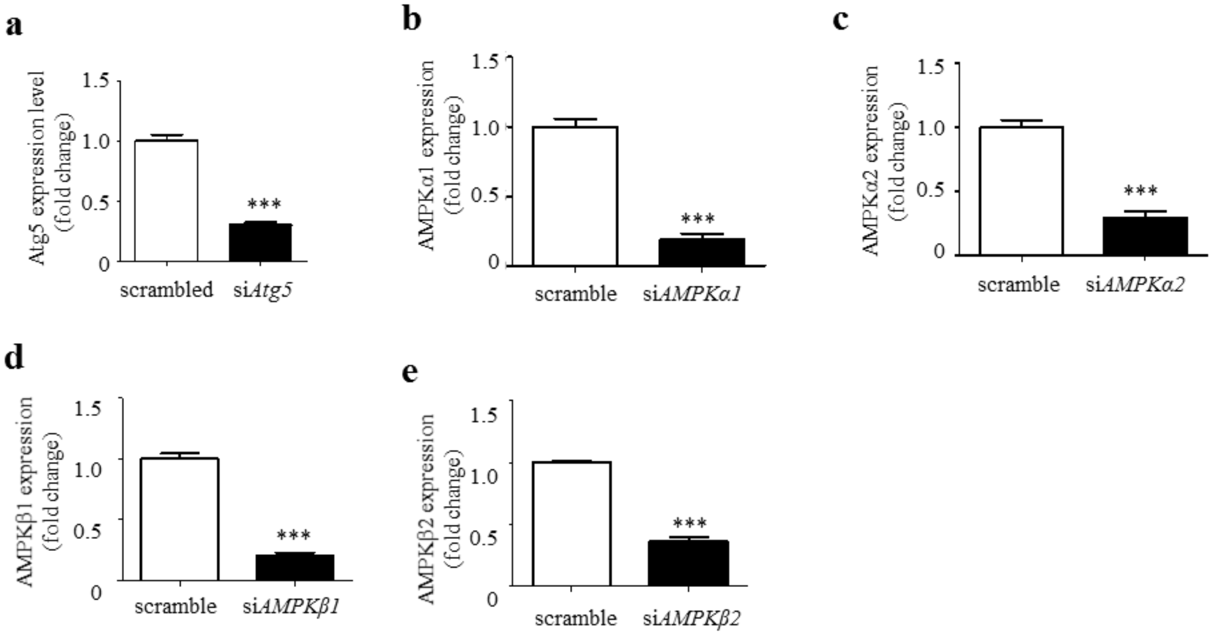

Supplemental Fig. S2. (**a**) Quantitation of Atg5 protein expression in scrambled and si*Atg5* cells. Data represented means ± S.D., $n = 6$. ***$P < 0.001$ scrambled versus si*Atg5* cells. (**b**) Quantitation of AMPKα1 protein expression in scrambled and si*AMPKα1* cells. Data represented means ± S.D., $n = 6$. ***$P < 0.001$ scrambled versus si*AMPKα1* cells. (**c**) Quantitation of AMPKα2 protein expression in scrambled and si*AMPKα2* cells. Data represented means ± S.D., $n = 6$. ***$P < 0.001$ scrambled versus si*AMPKα2* cells. (**d**) Quantitation of AMPKβ1 protein expression in scrambled and si*AMPKβ1* cells. Data represented means ± S.D., $n = 6$. ***$P < 0.001$ scrambled versus si*AMPKβ1* cells. (**e**) Quantitation of AMPKβ2 protein expression in scrambled and si*AMPKβ2* cells. Data represented means ± S.D., $n = 6$. ***$P < 0.001$ scrambled versus si*AMPKβ2* cells.

Supplemental Fig. S3

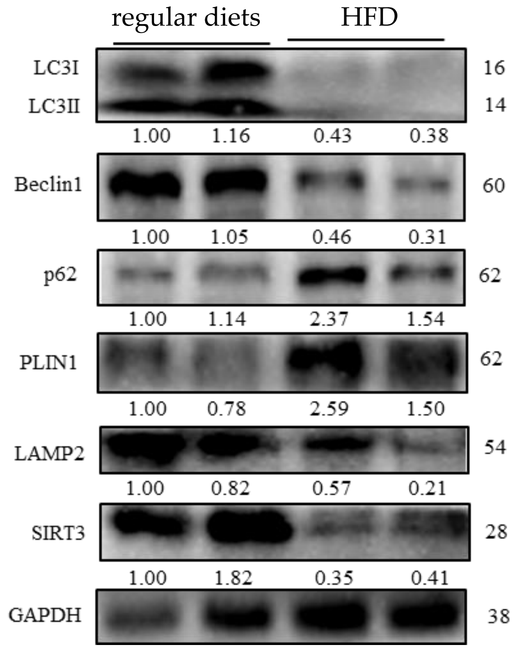

Supplemental Fig. S3. The expression of LC3, Beclin1, p62, PLIN1 and LAMP2A in epididymal WAT from regular diet- and HFD-fed mice. GAPDH was used as a loading control.

Supplemental Fig. S4

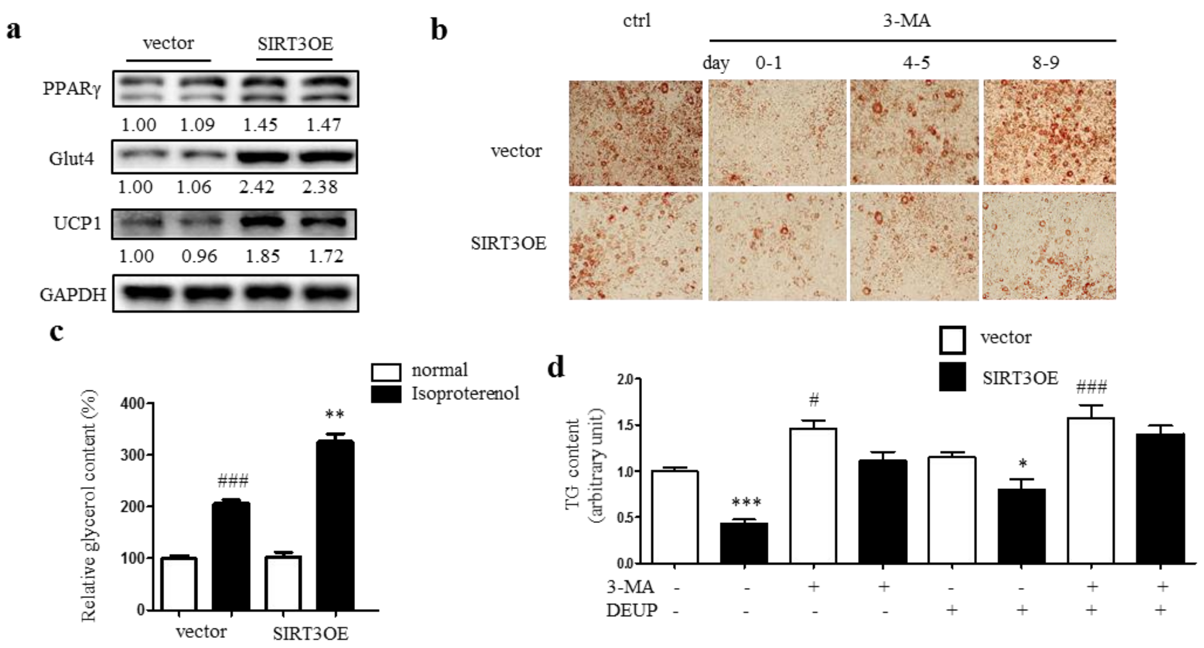

Supplemental Fig. S4. (**a**) The expression of PPARγ, GLUT4 and UCP1 in SIRT3OE and vector cells. (**b**) The vector and SIRT3OE adipocytes were treated with DMSO (ctrl) or 3-MA (at day 0, 6, and 8, respectively) for 24 h. Oil Red O staining was performed on day 10 post differentiation. (**c**) Relative glycerol content in vector and SIRT3OE adipocytes under both normal and isoproterenol-induced conditions. White: normal; black: isoproterenol. $^{**}P < 0.01$, vector versus SIRT3OE cells; $\#\#\#P < 0.001$ normal versus isoproterenol treatment. (**d**) TG content in vector and SIRT3OE adipocytes. After fully differentiation, cells were treated with DMSO (vehicle control), 3-MA, DEUP, or combination of 3-MA and DEUP for 24 h. $^{*}P < 0.05$ and $^{***}P < 0.001$ vector versus SIRT3OE cells; $\#P < 0.05$ and $\#\#\#P < 0.001$ vehicle versus treated cells. Data represented means ± S.D., $n = 6$.

Supplemental Fig. S5

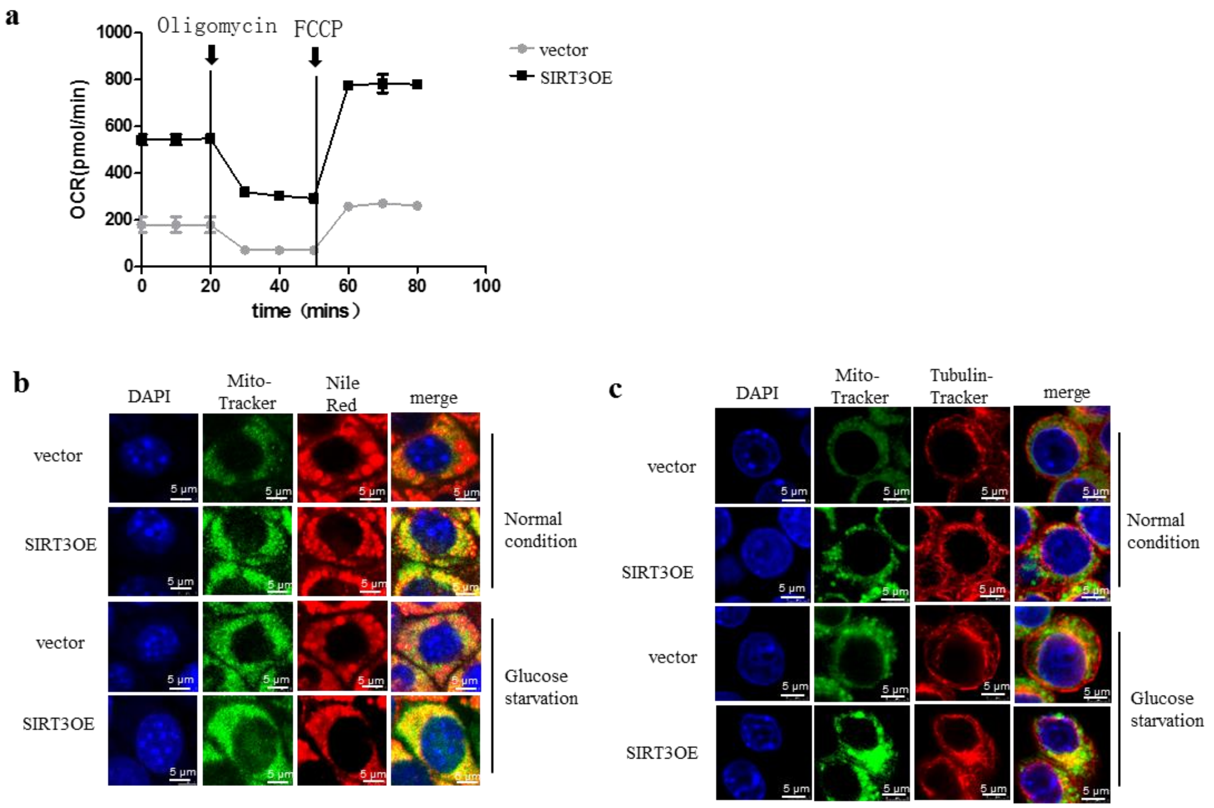

Supplemental Fig. S5 SIRT3 promotes LD–mitochondria interaction with detyrosinated microtubules under nutrient starvation. (**a**) OCR of vector and SIRT3OE cells under baseline and oligomycin and FCCP induction condition. (b) Confocal images of vector and SIRT3OE cells under normal condition or glucose starvation status. Nile red (red) and mitotracker (green). Scale bar = 5 μm. (**c**) Confocal images of vector and SIRT3OE cells under normal condition or glucose starvation status. Mitotracker (green) and tubulin Tracker (red). Scale bar = 5 μm.
